# Mimicking platelet indices in patients with malaria and dengue hemorrhagic fever: characteristics and clinical applications

**DOI:** 10.1186/s41182-022-00467-8

**Published:** 2022-10-11

**Authors:** Nant The Su Mon, Noppadon Tangpukdee, Prakaykaew Charunwatthana, Kobporn Boonnak, Srivicha Krudsood, Shigeyuki Kano, Polrat Wilairatana, Wattana Leowattana

**Affiliations:** 1 Department of Clinical Tropical Medicine, Faculty of Tropical Medicine, Mahidol University, 420/6 Ratchawithi Road, Bangkok, Thailand; 2 Mahidol Oxford Research Unit, Faculty of Tropical Medicine, Mahidol University, Bangkok, Thailand; 3 Department of Immunology, Faculty of Medicine Siriraj Hospital, Mahidol University, Bangkok, Thailand; 4 Department of Microbiology and Immunology, Faculty of Tropical Medicine, Mahidol University, Bangkok, Thailand; 5 Department of Tropical Hygiene, Faculty of Tropical Medicine, Mahidol University, Bangkok, Thailand; 6 Department of Tropical Medicine and Malaria, Research Institute, National Center for Global Health and Medicine, Tokyo, Japan; 7 WHO Collaborating Centre for Case Management, Training and Research on Malaria, Faculty of Tropical Medicine, Mahidol University, Bangkok, Thailand

**Keywords:** *Plasmodium falciparum* malaria, *Plasmodium vivax* malaria, Dengue hemorrhagic fever, Platelet indices, Potential prognostic marker, Thailand

## Abstract

**Background:**

Although platelet indices are routinely available using automated blood cell counters, the clinical applications of these parameters for malaria and dengue hemorrhagic fever (DHF) have not been substantially implemented. We conducted this study to investigate the potential role of platelet indices as a prognostic marker in adult patients with *Plasmodium vivax* malaria, *Plasmodium falciparum* malaria, and DHF admitted to the Hospital for Tropical Diseases, Bangkok, Thailand.

**Methods:**

We enrolled 219 eligible patients, comprising 96 with *P. falciparum* malaria, 71 with *P. vivax* malaria, and 52 with DHF. We evaluated the study groups’ baseline clinical features and alterations of platelet indices during the first 4 days of admission.

**Results:**

Upon admission, the initial laboratory findings showed no statistically significant difference in platelet count (PC), plateletcrit (PCT), or platelet distribution width (PDW) between patients with *P. vivax* and *P. falciparum*; however, mean platelet volume (MPV) was significantly higher in patients with *P. falciparum*. Comparisons of the initial platelet indices in malaria and DHF showed that only PC and PCT were significantly lower in DHF. Although MPV in DHF tended to be lower than in malaria, a statistically significant difference was observed only with *P. falciparum*. Moreover, the results also showed no significant alterations in the platelet indices among the study groups during the first 4 days of admission.

**Conclusions and recommendations:**

Clinical presentations of DHF and malaria are nonspecific and may overlap with other common tropical diseases. Alterations of initial platelet indices may be investigated in *P. vivax* and *P. falciparum* malaria mimicking DHF. Although a significant reduction in PC and PCT in DHF might be a clue for differential diagnosis of malaria, the use of MPV and PDW might be impractical. We suggest that appropriate laboratory diagnoses for malaria and dengue infections are still needed for the differential diagnosis of acute febrile patients who have a risk of malaria or dengue infections. To clarify the clinical utility of platelet indices in patients with dengue and malaria, further studies are required that particularly include patients with different severities, geographical areas, and levels of health care settings.

## Background

Malaria and dengue hemorrhagic fever (DHF) are important mosquito-borne diseases caused by parasites and viruses that are transmitted to people via the bites of infected *Anopheles* and *Aedes* mosquitoes, respectively. Malaria and DHF are life-threatening diseases affecting subtropical and tropical countries, particularly Southeast Asia and Asia–Pacific regions [1].

A 2021 report by the World Health Organization (WHO) estimated that almost one-half of people worldwide are at risk of malaria. There were about 14 million more patients with malaria in 2020 as compared with 2019, and ~ 47,000 of these lethal cases (two-thirds of the 69,000 more deaths in 2020 as compared with 2019) might be attributed to disruptions in health care services during the COVID-19 pandemic. The African region still continues to carry a high proportion of the global malaria burden, with 95% of all malaria patients and about 95% of all deaths in the year 2020, and children younger than 5 years account for about 80% of all malaria deaths [2]. Malaria remains a serious infectious disease in many parts of Asia, and the Southeast Asia region has the second highest estimated malaria burden globally [3].

The global burden of dengue has been noticed, as the global incidence of the disease has risen dramatically in recent decades. Evidence also shows that nearly half of the global population is at risk of dengue infection. A current report indicated that the number of dengue patients has been increasing in many countries around the world, including Thailand [4].

Platelets, anucleate blood cells, play important roles in thrombosis, hemostasis, and inflammatory mechanisms. Clinical studies have indicated the important role of platelets in the pathogenesis of the clumping of parasitized red blood cells in severe malaria and bleeding in dengue infection [5–8]. Although previous investigations demonstrated thrombocytopenia in malaria and dengue patients, few studies focused on alterations of platelet indices. Platelet indices, biomarkers of platelet activation, can be estimated at a low cost using automated blood cell counters, which are available in routine laboratories. Recently, the significance of platelet indices has been noticed in the clinical investigation of various diseases [9]. However, the significance and application of platelet indices for the clinical management of patients with malaria and dengue have not been substantially examined. We demonstrate the characteristics and alterations of initial platelet indices to investigate the potential role as a prognostic marker in adult patients admitted with *Plasmodium vivax* malaria, *Plasmodium falciparum* malaria, and DHF in the tertiary care setting, in Thailand. We hope that our findings may be helpful for clinicians who are working in endemic areas of malaria and dengue infections.

## Materials and methods

### Study site and enrollment procedures

This study was conducted with approval at the Hospital for Tropical Diseases, Faculty of Tropical Medicine, Mahidol University, a tertiary care setting associated with a medical school, located in Bangkok, Thailand, for the clinical management of common tropical diseases, particularly dengue and malaria infections. The hospital is well-equipped for complicated case management (e.g., patients with severe malaria or dengue). The protocol was reviewed and certified by the institutional review board before we conducted the study. To secure participants’ confidentiality and to protect participants’ privacy, all identifications relating to patients were recorded as anonymous on all research documents and in the database. Upon admission, preinclusion criteria for malaria patients were patients presenting with positive blood films, confirmed by the microscopic method, for acute *P. falciparum* malaria monoinfection or acute *P. vivax* malaria monoinfection. Preinclusion criteria for DHF patients were patients showing positive diagnostic results for dengue infection, confirmed by rapid immunochromatographic diagnosis for dengue NS1 antigen or anti-dengue immunoglobulin M (IgM) or immunoglobulin G (IgG). All patients who fulfilled the inclusion–exclusion criteria were enrolled in the study. The inclusion criteria were (1) either female or male, (2) age ≥ 15 years, (3) body weight ≥ 40 kg, (4) ability to remain in the hospital for at least 4 days after starting treatments, and (5) no history of underlying diseases of hematological disorders. We excluded lactating or pregnant women, patients with mixed malaria infections or co-infection of any infectious diseases, and patients with significant systemic diseases requiring specific therapy (e.g., immunosuppressive disorders, malignancy, metabolic, gastrointestinal, endocrine, neurologic, endocrine, pulmonary, or cardiovascular diseases).

### Specific and symptomatic treatments

Artemisinin-based combination therapies were prescribed for patients with *P. falciparum* malaria. Patients with *P. vivax* malaria were treated with chloroquine and primaquine. Apart from specific treatment with antimalarial regimens, supportive and symptomatic treatments were provided for study patients, including patients with DHF following standard care at the Hospital for Tropical Disease, Bangkok, Thailand.

### Laboratory and clinical data collection

Laboratory and clinical variables were extracted according to the case record form. Parameters consisted of gender, age, occupations, underlying diseases, initial vital signs, and laboratory investigations for platelet indices before and after treatment (the first 4 days of admission). We used an automated blood cell counter (Advia 120 Hematology System commercially serviced by Siemens Medical Solutions Diagnostics and reagents commercially served by Roche Diagnostics, Berlin, Germany) to determine platelet indices (e.g., platelet count [PC]; normal range 150–450 × 10^3^/µL), mean platelet volume (MPV; normal range 7.2–11.1 fL), platelet distribution width (PDW; normal range 25–65%), and plateletcrit (PCT; normal range 0.12–0.36%). Commercial products to investigate anti-dengue IgM or IgG and dengue NS1 antigen (SD BIOLINE Dengue Duo; Standard Diagnosis, Republic of Korea) were applied for the diagnosis of dengue infection. We applied thin and thick blood smear techniques using the Giemsa stain method for malaria diagnosis. Clinical definitions of malaria and dengue infections followed WHO criteria [10, 11].

### Statistical analysis

Qualitative and quantitative variables were analyzed. We used two-tailed testing for reporting all *p* values and assigned 0.05 to indicate a statistically significant difference. Normality testing was based on the Kolmogorov–Smirnov test. We found that the distribution of data generally did not present normality. Therefore, the data were presented as median (range) and frequency of observations. The Chi-square test or Fisher’s exact test was used for testing categorical data, as appropriate. The differences between quantitative variables were tested by Mann–Whitney *U* test. Kruskal–Wallis test was applied for testing the differences among quantitative variables, and Friedman’s test was used to evaluate differences in each item of the platelet indices during the first 4 days of admission.

## Results

### General baseline clinical description

Upon admission, a total of 219 eligible cases who fulfilled all inclusion–exclusion criteria were enrolled as follows: 71 patients had *P. vivax* malaria infection, 96 patients had *P. falciparum* malaria infection, and 52 patients had DHF. The baseline clinical characteristics among the three study groups are presented in Table 1. Both malaria groups had a median age of 27 years (range 15–64 years), and the median age was 24 years (range 16–73 years) in the DHF group. There were 80 (83.3%), 65 (91.5%), and 27 (52.0%) male patients in the *P. falciparum*, *P. vivax*, and DHF groups, respectively. Fever (core body temperature ≥ 37.5 °C) was found to be the most common manifestation in study patients, followed by chill-rigor (89.6%), headache (80.2%), and myalgia (61.5%) in patients with *P. falciparum* malaria; chill-rigor (90.1%), myalgia (76.1%), and headache (53.5%) in patients with *P. vivax* malaria; and myalgia (65.4%), rash (57.7%), nausea/vomiting (36.5%), and chill-rigor (34.6%) in the DHF group. According to the treatment schedule, no major adverse event or fatality occurred during the study.

**Table 1:** Baseline clinical characteristics of study patients on admission

	Baseline data	*P. falciparum* patients (*n* = 96)	*P. vivax* patients (*n* = 71)	DHF patients (*n* = 52)	*p* values
Characteristics	Gender [Male: *n* (%)]	80 (83.3)	65 (91.5)	27 (52.0)	< 0.001
	Age (years) Median (range)	27 (15–64)	27 (15–64)	24 (16–73)	0.550
Occupations *n* (%)	Laborer	58 (60.4)	44 (62.0)	13 (25.0)	< 0.001
	Student	6 (6.3)	1 (1.4)	13 (25.0)	
	Office worker	4 (4.2)	2 (2.8)	8 (15.4)	
	Others^a^	25 (26.0)	17 (23.9)	9 (17.3)	
	Unemployed	3 (3.1)	7 (9.9)	9 (17.3)	
Initial vital signs Median (range)	Body core temperature (°C)	37.9 (36.0–40.0)	37.8 (36.4–40.2)	37.5 (36.0–40.9)	0.178
	Systolic blood (mmHg)	110 (84–156)	112 (89–165)	111 (80–143)	0.962
	Diastolic blood (mmHg)	69 (42–100)	66 (45–96)	70 (46–93)	0.723
	Pulse rate (/min)	92 (60–133)	92 (60–142)	88 (52–120)	0.091
	Respiratory rate (/min)	22 (16–42)	20 (18–26)	20 (14–28)	< 0.001
Clinical presentations *n* (%)	Chill and rigor	86 (89.6)	64 (90.1)	18 (34.6)	< 0.001
	Headache	77 (80.2)	38 (53.5)	17 (32.7)	< 0.001
	Myalgia	59 (61.5)	54(76.1)	34 (65.4)	0.133
	Nausea/vomiting	50 (52.1)	18 (25.4)	19 (36.5)	0.002
	Abdominal discomfort	18 (18.8)	7 (9.9)	16 (30.8)	0.013
	Diarrhea	13 (13.5)	13 (18.3)	12 (23.1)	0.332
	Cough	36 (37.5)	16 (22.5)	8 (15.4)	0.008
	Rash	1 (1.0)	0	30 (57.7)	< 0.001

^a^Other jobs included monk, housewife, shopkeeper, seller, mechanic, driver, and gardener

### Comparisons of initial platelet indices in different disease groups

**Fig. 1:**
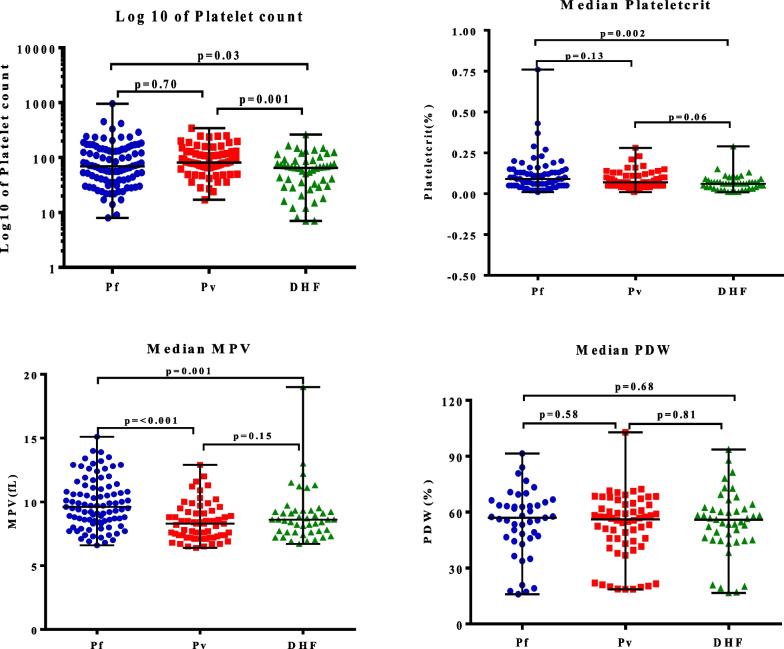
Initial platelet indices in the different disease groups on admission. *Pf* patients with *P. falciparum*; *Pv* patients with *P. vivax*; *DHF* patients with dengue hemorrhagic fever; *MPV* mean platelet volume; *PDW* platelet distribution width

Upon admission, initial platelet indices were demonstrated to compare the differences among the three study groups. Initial laboratory results did not show statistical significance in PC, PCT, or PDW levels between patients with *P. vivax* and *P. falciparum*. However, we found that the initial MPV of *P. vivax* patients was significantly lower than in *P. falciparum* patients. Comparative studies of platelet indices in DHF and malaria patients showed that only PC and PCT were statistically significantly different in DHF patients. It was interesting that MPV in patients with DHF tended to be lower than in malaria patients, but statistical significance was observed only in patients with *P. falciparum*. In addition, we found no statistically significant differences in initial PDW among the three study groups (Fig. 1).

### Alterations in platelet indices during the first 4 days of admission

**Fig. 2:**
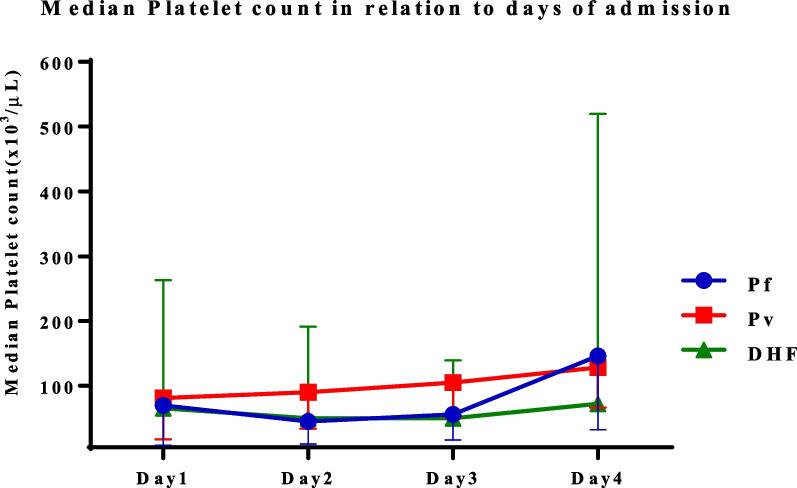
Median platelet count in the different disease groups from day 1 to day 4 of admission

Alterations in platelet indices among patients with *P. falciparum*, *P. vivax,* and DHF were evaluated in the first 4 days after admission. We found a decrease in the median PC on day 1, and the lowest level was observed in patients with DHF (65,000/μL) as compared with patients with *P. falciparum* (69,500/μL) and *P. vivax* (81,000/μL). We noted that the median PC gradually declined in patients with *P. falciparum* and DHF until day 3; after that, the level returned to normal on day 4 for patients with *P. falciparum*. However, this was not observed in DHF patients, because the baseline PC might be lower than its correspondences [*P. falciparum, P. vivax*, and the normal range (lowest)]. In patients with *P. vivax*, the median PC tended to increase up to day 4 after admission, compared with the baseline level. Only the median PC of patients with *P. falciparum* reached a normal level (150 × 10^3^/μL) within 4 days after admission (Fig. 2).

**Fig. 3:**
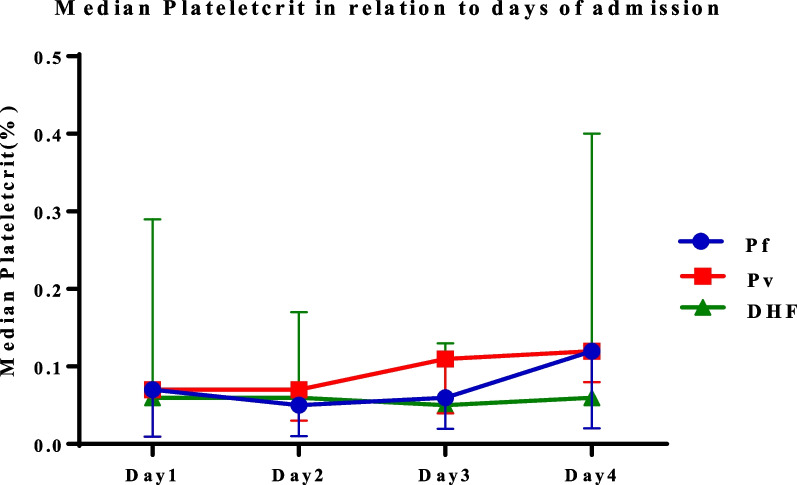
Median plateletcrit in the different disease groups from day 1 to day 4 of admission

A similar pattern was observed in the dynamicity of PCT (Fig. 3). We noted that the median PCT of *P. vivax* and *P. falciparum* cases was found to be equal on day 1 (0.07%), but a lower value was observed in patients with DHF (0.06%). The median PCT of *P. falciparum* decreased to 0.05% on day 2. However, on day 3, the median PCT increased to 0.06% and continually increased to 0.12% on day 4. With regard to patients with *P. vivax*, an increasing trend in the median PCT was observed during the first 4 days after admission. The median PCT on day 1 and day 2 was equal at 0.07% and increased to 0.11% on day 3 and 0.12% on day 4, respectively. We also found that the median PCT in DHF tended to be constant during the study period. The median levels on day 1 and day 2 were 0.06%. The level on day 3 declined slightly to 0.05%, and then the value returned to 0.06% on day 4.

**Fig. 4:**
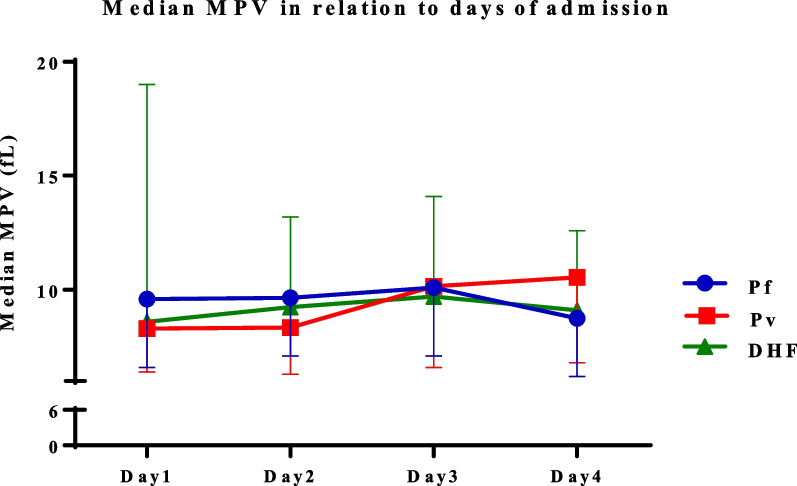
Median mean platelet volume in different disease groups from day 1 to day 4 of admission

Interestingly, a negative trend was observed in the median MPV during the study period (Fig. 4). On day 1, the median MPV of *P. vivax* (8.3 fL) and DHF (8.6 fL) was slightly lower than in *P. falciparum* malaria patients (9.6 fL). The median values in *P. falciparum* malaria patients seemed to be constant on days 1, 2, and 3 and then dropped slightly to 8.75 fL on the fourth day. The median MPV in patients with *P. vivax* was constant on day 1 (8.3 fL) and 2 (8.35 fL) and then raised up to 10.15 fL on day 3 and 10.55 fL on day 4, respectively. In patients with DHF, an increasing trend in the median MPV was found on day 2 and day 3 (9.25 fL and 9.7 fL), respectively. However, the median level declined slightly on day 4 (9.1 fL).

**Fig. 5:**
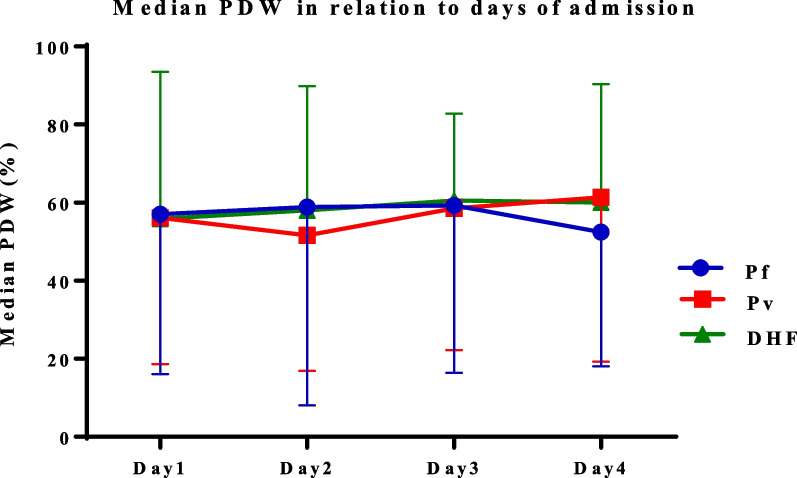
Median platelet distribution width value in the different disease groups from day 1 to day 4 of admission

The dynamicity of median PDW was also investigated in the different study groups. On the first day, the median PDW values in patients with *P. falciparum* (57%), *P. vivax* (56.1%), and DHF (55.9%) were closely presented. In *P. falciparum*, an increasing level was observed on day 3 (59.25%) but decreased to 52.45% on day 4. The value of *P. vivax*, however, was decreased to 51.6% and then increased up to 61.4% on day 2 and day 4, respectively. Interestingly, the median PDW in patients with DHF increased to 60.5% on day 3 and then decreased to 60% on the fourth day (Fig. 5). Unfortunately, statistically significant differences in the platelet indices’ alterations during the first 4 days of hospitalization could not be observed, either between or within study groups.

## Discussion

Despite global progress in clinical and laboratory infectious research, tropical and vector-borne diseases continue to pose a significant burden of disease worldwide [1]. Dengue and malaria infections remain public health problems in many countries, including Thailand [3, 4]. Hematologic abnormalities are well-known common clinical complications of these diseases and are frequently observed in patients with dengue and malaria infections. These complications also play important roles in disease severity and fatality. It was reported that the risk of hematologic alterations, including thrombocytopenia, in dengue and malaria infections was associated with various clinical factors (e.g., immunity status, level of endemicity, demographic factors, individual hemoglobinopathy, and nutritional status of patients) [6, 8]. In this study, we investigated platelet indices (e.g., PC, PCT, MPV, and PDW) to illustrate alteration patterns in patients with dengue and malaria that could be implied as a prognostic clue in the clinical differential diagnosis.

The clinical presentations of patients with dengue and malaria may mimic and overlap those of many other common infectious tropical diseases. Here, we noticed that the major clinical features of *P. vivax* and *P. falciparum* malaria patients were fever, chills and rigor, headache, and myalgia. Our study results were consistent with those of Chhong et al.’s, who noted that fever, myalgia, headache, and rash were common manifestations of DHF patients [12]. Although mild abnormal bleeding was observed in some patients with DHF, mucosal bleeding signs were the most common finding, including gum bleeding, epistaxis, and petechial hemorrhage. Our findings were consistent with previous reports indicating that this complication might be associated with thrombocytopenia [13, 14]. Interestingly, our results showed that male patients predominated among patients with dengue and malaria infection. This finding might possibly be explained by the places, where the patients were employed or by their history of traveling to endemic areas, as described by Smith et al. [15]. However, the findings of a recent study do not support our results that males were more likely to be exposed to malaria infections than females [16]. Therefore, further studies on the social–behavioral aspects of the disease should be investigated to clarify this evidence in different geographical areas.

Thrombocytopenia (PC < 150 × 10^3^/µL) is a clinical laboratory condition that is usually observed in patients suffering from dengue infection and *P. falciparum* and *P. vivax* malaria. However, severe bleeding and platelet transfusion are rarely required [12–14, 17–20]. Peripheral platelet destruction, platelet sequestration, and bone marrow suppression are postulated as the possible mechanisms of this condition in patients with malaria and dengue [5, 6, 8, 21]. Our findings of low PC on day 1 concur with the results of other studies. We also noted that the frequency of thrombocytopenia was approximately 78%, 80%, and 90% in patients with *P. falciparum*, *P. vivax*, and DHF, respectively. In our findings, a reduction in the patterns of PC did not differ statistically among the different disease groups, leading to the assumption that similar mechanisms govern this condition. With regard to thrombocytopenia and coagulation abnormality conditions in patients with dengue and malaria, most patients with DHF in our study had a PC level of < 150 × 10^3^/µL and an initial median PC that was statistically lower than in patients who were infected with malaria. In patients with DHF, up to 38% presented with a PC level of < 50,000/µL, and about 26.9% of the study patients were clinically associated with mild abnormal mucosal bleeding. This might be due to the intrinsic decrease in platelet values in adult patients superimposed on the bone marrow’s direct effect of the dengue virus. Other reasons could be the alteration in cytokines, which interferes with the function of the bone marrow, or the immune-mediated destruction of platelets rather than platelet sequestration [22]. However, our study results might have been affected by the inclusion criteria, as we enrolled only patients with DHF, and coagulation data (to determine the causes of bleeding) were available for only a few of the included patients.

At present, platelet indices can be investigated and estimated at a low cost using common hematological analyzers that are available in the laboratory units of general hospitals. Current reports have described the role of platelet indices (e.g., PCT, MPV, or PDW) as potential laboratory markers in patients suspected of having malaria or dengue [6, 18, 23, 24]. Although platelet indices have been considered to be important biological markers, they are discerned to be less important and are not used widely in clinical practice for managing infectious diseases, including dengue and malaria [6, 25, 26].

PDW is a laboratory marker of platelet anisocytosis, indicating variability in the volume and size distribution of platelets. The average size of platelets may be indicated by the MPV levels for clinical bone marrow activity. Total platelet biomass could be implied by the PCT value, showing a percentage of platelet volume occupied in blood. Therefore, PCT might be useful as a screening tool for detecting a quantitative platelet abnormality, because it is a combination between MPV and PC [9, 27]. According to our findings, we noted that about 70% of malaria patients had low PCT. This finding might be explained by the combination of low PC with high MPV (24%), low PC with low MPV (8%), low PC with normal MPV (64%), and normal PC with low MPV (4%). This might also imply that a reduction in the PCT of patients might result from a decrease in PC rather than platelet size. Interestingly, in patients with DHF, we found that the initial PCT was statistically lower than in malaria patients, although some values were presented within the normal range. The possible mechanism for this might be the decreasing size and number of platelets in patients with DHF. Although the results in DHF patients were not significant, the median MPV tended to be lower as compared with malaria patients. In malaria patients, the compensation of bone marrow to low platelet levels at peripheral blood circulation and the mechanism of platelet activation might be possible explanations for the higher MPV levels. In DHF, interference of bone marrow function might occur due to the cytokines, induced by the dengue virus or as a direct effect of the dengue virus itself, and the partial failure of clinical compensation during PC reduction. Another clinical mechanism of MPV reduction in DHF is the selective consumption of platelets during abnormal bleeding signs. This could possibly explain the MPV reduction in patients with DHF as compared with patients with malaria. Moreover, MPV might be associated with thrombocytopenia, and a reduction in MPV might be clinically correlated with abnormal bleeding in DHF patients presenting with thrombocytopenia [22, 24, 27]. In addition, increasing levels of PDW and MPV might result from platelet activation, as presented in various diseases (e.g., cardiovascular and cerebrovascular diseases, sepsis, tuberculosis, or inflammatory disorders) [28, 29]. We concur with Bayleyegn that an increase in MPV and PDW with a decrease in PCT and PC might be a predictor of the disease severity of patients with malaria [6]. Our results also imply that the activation of systemic endothelial cells with platelet sequestration in the peripheral blood circulation of malaria patients as well as the platelet aggregates and clinical formation of mega platelets as giant platelets were detected almost 50% of malaria patients. The elevation of PDW values could also imply that destruction, swelling, or immaturity of platelets may cause an enlargement in the platelet size. It was also suggested that another possible explanation for the high value of PDW could be a greater variation in the heterogeneity in the platelet sizes in patients with malaria as compared with patients with DHF, and the clinical relationship between PDW and MPV was not as varied as in healthy subjects [24, 27].

Because platelet indices are machine specific, in cases of thrombocytopenia, these indices might not be reported in blood specimens in some machines. Moreover, this result of platelet indices might be interfered with by abnormal red blood cell parameters, such as in patients with thalassemia disease, who were not included in this study. Another limitation of this study was the days of admission at the early stage. The day of admission might not reflect patients’ true thrombocytopenia-related condition, particularly in patients with dengue infection, as they might be in different infection phases (febrile versus toxic phases), which could affect PC and platelet indices. Moreover, changes in platelet parameters are dynamic; therefore, an analysis of the delta change in the platelet parameters might better explain the patient’s condition, and a combination of platelet parameters might provide a more complete picture of the patient for further investigation.

In conclusion, our findings show that alteration patterns in platelet indices, between DHF and malaria, might be impractical as a potential laboratory biomarker for clinical differential diagnosis. Although we found a significant reduction in the PC and PCT of patients with DHF, PDW and MPV did not demonstrate significance in comparison with malaria patients. Therefore, differential diagnosis using platelet indices as a clinical laboratory predictor in patients with dengue and malaria should not be used for clinical practice. We suggest that malaria should be microscopically detected and an appropriate laboratory diagnosis obtained for dengue infection for differential diagnosis, particularly in acute febrile patients who are at risk of malaria and dengue infections. Because both malaria and dengue infections share the same pathophysiology, the use of a PC and platelet indices might be more beneficial for determining the severity of the disease than the differential diagnosis of both diseases separately. Morbidity and mortality in patients with malaria and dengue could be reduced by appropriate prompt treatment after an early effective diagnosis. Therefore, further studies of platelet indices in patients with dengue and malaria, particularly among those with conditions of different severity, different geographical areas, and different levels of health care settings, may be required to construct practical measures for these patients.

## Data Availability

The clinical data set and analyzed results of the study will be available for reasonable request.

## References

[1] World Health Organization. Vector-borne diseases. 2020. https://www.who.int/news-room/fact-sheets/detail/vector-borne-diseases. Accessed 15 Mar 2022.

[2] World Health Organization. World malaria report 2021. 2021. https://www.who.int/teams/global-malaria-programme/reports/world-malaria-report-2021. Accessed 15 Mar 2022.

[3] World Health Organization. Malaria in South-East Asia. 2022. https://www.who.int/southeastasia/health-topics/malaria. Accessed 15 Mar 2022.

[4] World Health Organization. Dengue and severe dengue. 2022. https://www.who.int/news-room/fact-sheets/detail/dengue-and-severe-dengue. Accessed 15 Mar 2022.

[5] Mayor A, Hafiz A, Bassat Q, Rovira-Vallbona E, Sanz S, Machevo S (2011). Association of severe malaria outcomes with platelet-mediated clumping and adhesion to a novel host receptor. PLoS ONE.

[6] Bayleyegn B, Asrie F, Yalew A, Woldu B (2021). Role of platelet indices as a potential marker for malaria severity. J Parasitol Res.

[7] Srichaikul T, Nimmannitya S, Sripaisarn T, Kamolsilpa M, Pulgate C (1989). Platelet function during the acute phase of dengue hemorrhagic fever. Southeast Asian J Trop Med Public Health.

[8] Archuleta S, Chia PY, Wei Y, Syed-Omar SF, Low JG, Oh HM (2020). Predictors and clinical outcomes of poor platelet recovery in adult dengue with thrombocytopenia: a multicenter, prospective study. Clin Infect Dis.

[9] Pogorzelska K, Kretowska A, Krawczuk-Rybak M, Sawicka-Zukowska M (2020). Characteristics of platelet indices and their prognostic significance in selected medical condition—a systematic review. Adv Med Sci.

[10] World Health Organization. Guidelines for the treatment of malaria. 2015. https://apps.who.int/iris/handle/10665/162441. Accessed 15 Mar 2022.

[11] World Health Organization. Comprehensive guidelines for prevention and control of dengue and dengue haemorrhagic fever, revised and expanded edition. 2011. https://apps.who.int/iris/handle/10665/204894. Accessed 15 Mar 2022.

[12] Chhong LN, Poovorawan K, Hanboonkunupakarn B, Phumratanaprapin W, Soonthornworasiri N, Kittitrakul C (2020). Prevalence and clinical manifestations of dengue in older patients in Bangkok Hospital for tropical diseases, Thailand. Trans R Soc Trop Med Hyg.

[13] Leowattana W, Tangpukdee N, Thar SK, Nakasiri S, Srivilairit S, Kano S (2010). Changes in platelet count in uncomplicated and severe falciparum malaria. Southeast Asian J Trop Med Public Health.

[14] Hottz E, Tolley ND, Zimmerman GA, Weyrich AS, Bozza FA (2011). Platelets in dengue infection. Drug Discov Today Dis Mech.

[15] Smith JL, Auala J, Haindongo E, Uusiku P, Gosling R, Kleinschmidt I (2017). Malaria risk in young male travellers but local transmission persists: a case–control study in low transmission Namibia. Malar J.

[16] Quaresima V, Agbenyega T, Oppong B, Awunyo JADA, AduAdomah P, Enty E (2021). Are malaria risk factors based on gender? A mixed-methods survey in an urban setting in Ghana. Trop Med Infect Dis.

[17] Wilairatana P, Tangpukdee N, Muangnoicharoen S, Krudsood S, Kano S (2010). Atypical lymphocytes in malaria mimicking dengue infection in Thailand. Res Rep Trop Med.

[18] Jalaly F, Jalaly T (2018). Can Platelet count helps in suspecting malaria infection? Data analysis from tertiary care hospital. Med Int J Pathol.

[19] Punnath K, Dayanand KK, Chandrashekar VN, Achur RN, Kakkilaya SB, Ghosh SK (2019). Association between inflammatory cytokine levels and thrombocytopenia during *Plasmodium falciparum* and *P. vivax* infections in south-western coastal region of India. Malar Res Treat..

[20] Awoke N, Arota A (2019). Profiles of hematological parameters in *Plasmodium falciparum* and *Plasmodium vivax* malaria patients attending Tercha General Hospital, Dawuro Zone, South Ethiopia. Infect Drug Resist.

[21] Quirino-Teixeira AC, Andrade FB, Pinheiro MBM, Rozini SV, Hottz ED (2022). Platelets in dengue infection: more than a numbers game. Platelets.

[22] Assinger A (2014). Platelets and infection—an emerging role of platelets in viral infection. Front Immunol.

[23] Navya BN, Patil S, Kariappa TM (2016). Role of platelet parameters in dengue positive cases—an observational study. Int J Health Sci Res.

[24] Wayez A, Zafar L, Aijaz M, Afroz N (2020). Study of platelet indices in dengue fever with thrombocytopenia and correlation of immature platelet fraction (IPF) with platelet recovery. Arch Hematol Case Rep Rev.

[25] Bashir AB, Saeed OK, Ageep AK (2015). Role of platelet indices in patients with dengue infection in Red Sea State Sudan. Int J Sci Res.

[26] Efe S, Asker I, Volkan I (2019). Prognostic significance of critical patients’ platelet indexes in mixed type critical care unit. J Med Surg Intensive Care Med.

[27] Vinholt PJ, Hvas AM, Nybo M (2014). An overview of platelet indices and methods for evaluating platelet function in thrombocytopenic patients. Eur J Haematol.

[28] Sahin F, Yazar E, Yıldız P (2012). Prominent features of platelet count, plateletcrit, mean platelet volume and platelet distribution width in pulmonary tuberculosis. Multidiscip Respir Med.

[29] Gao Y, Li Y, Yu X, Guo S, Ji X, Sun T (2015). The impact of various platelet indices as prognostic markers of septic shock. PLoS ONE.

